# Cognitive dysfunction in older patients undergoing non‐neurosurgery in the immediate postoperative period: A systematic review

**DOI:** 10.1002/nop2.70023

**Published:** 2024-08-27

**Authors:** Pinyapat Kitthanyateerakul, Thitipong Tankumpuan, Patricia M. Davidson

**Affiliations:** ^1^ Faculty of Nursing Mahidol University Bangkok Thailand; ^2^ The Vice‐Chancellor's Unit University of Wollongong Wollongong New South Wales Australia

**Keywords:** cognitive dysfunction, non‐neurosurgery, older, short‐term postoperative period, systematic review

## Abstract

**Aim:**

To explore the risk factors associated with postoperative cognitive dysfunction in older patients within the first 7 days after non‐neurosurgical surgery and anaesthesia.

**Design:**

A systematic review.

**Methods:**

Following, PRISMA 2020 (Preferred Reporting Items for Systematic Reviews and Meta‐analyses). Checklist, a systematic review of studies published from January 2018 to January 2024. The literature search was conducted across six electronic online databases, including PubMed, EMBASE, Scopus, Ovid, MEDLINE and Science Direct, and the Johns Hopkins Nursing Evidence‐Based Practice Evidence Rating Scale was used for study appraisal.

**Results:**

The initial search yielded 1750 studies. The review included 19 studies which comprised prospective observational, case–control and retrospective studies. The prevalence of postoperative cognitive dysfunction ranged from 19% to 64% among older adults undergoing non‐neurosurgery. The identified risk factors were classified into three phases including preoperative, intraoperative and postoperative. Preoperative risk factors were found in age, educational attainment, malnutrition, preoperative biomarkers and co‐morbidities. Intraoperative risk factors were the duration of the operation, blood loss during the operation and anaesthesia used. Postoperative risk factors consisted of postoperative biomarkers and postoperative pain.

**Patient or Public Contribution:**

The result from this review may assist researchers and healthcare providers in assessing the underlying causes and risk factors of postoperative cognitive dysfunction, and in formulating suitable preventative and therapeutic strategies for older adults with non‐neurosurgery during the short‐term postoperative period.

## 1 INTRODUCTION

Cognitive dysfunction, known as neurocognitive disorders, includes a wide range of intellectual abilities and processes, including attention, memory, knowledge, decision‐making, planning, reasoning, judgement, perception, comprehension, language and visuospatial function (Varpaei et al., 2024). A decline in the efficiency of neurological functions can be related to increasing age, neurological inflammation and trauma from neuromuscular diseases, brain tumours or abnormality of brain function (Tarumi & Zhang, 2018). Postoperative cognitive dysfunction (POCD) refers to a significant decline in cognitive function compared to the individual's presurgery baseline. Moreover, neurocognitive disorders that occur during the perioperative period is defined as disorders that include preoperative cognitive impairment, cognitive decline diagnosed within 1 month after surgery (referred to as delayed neurocognitive recovery) and cognitive decline diagnosed within 2–12 months after surgery (Evered et al., 2018; Evered & Silbert, 2018). The actual mechanisms, aetiology, predisposing factors and prevalence of POCD remain unclear with revealed incidence varying between 10% and 54% during the initial weeks following surgery, influenced by many factors, and is more common in older people. The complexity of risk‐stratifying individuals for POCD poses challenges in both the identification and institution of therapeutic approaches (Li et al., 2021).

Risk factors associated with the development of POCD have been established in multiple studies including patient‐related factors, surgery‐related factors and anaesthesia‐related factors (Lertkovit et al., 2022). Identification and diagnosis are a difficult task as it mostly involves a sustained deterioration in cognitive abilities that typically occurs after surgery and anaesthesia. Several studies have identified the risk factors and incidence of POCD that can manifest within 3 days following surgery. However, it is important to clearly distinguish cognitive dysfunction from postoperative delirium which typically occurs within the first 3 days after surgery and anaesthesia. In contrast, early cognitive changes may develop during the latter part of the first week after surgery and have the potential to persist for a longer duration (Glumac et al., 2019). The investigation of risk factors for POCD commenced some decades ago, resulting in the identification of certain factors that have gained widespread recognition, while others remain subjects of ongoing discussion and analysis. Identifying individuals at risk of cognitive impairment, particularly among older people undergoing non‐neurosurgery, is an important strategy to optimize patient outcomes in the immediate postoperative period. Therefore, the purpose of this review was to identify the risk factors for POCD in older patients undergoing non‐neurosurgery in a short‐term postoperative period of 7 days.

## 2 METHOD

### 2.1 Search strategy

We conducted this systematic review from January 2018 to January 2024. We utilized the Medical Subject Headings (MeSH) system to create relevant keywords for our search. The electronic databases PubMed, EMBASE, Scopus, Ovid, MEDLINE and Science Direct used the keywords “Postoperative Cognitive Dysfunction” OR “Postoperative Cognitive Decline” OR “Postoperative Cognitive Disorders.” Filters were used to access the online database to find full‐text articles using different study designs that were published in 2018 on older patients aged ≥60 years. The systematic review was conducted in accordance with the Preferred Reporting Items for Systematic Reviews and Meta‐analysis (PRISMA) checklist (Page et al., 2021).

### 2.2 Study selection

Inclusion criteria for the study were: (1) older patients undergoing surgery (non‐neurosurgery) with general anaesthesia both cardiac and non‐cardiac surgery; (2) the POCD was measured within 7 days after surgery. (3) The studies were conducted as case–control studies, cross‐sectional studies and cohort studies; (4) articles were published in English. The exclusion criteria were as follows: case reports, comments, reviews or other types of literature, unpublished master's thesis and doctoral dissertations.

### 2.3 Screening, extraction and quality appraisal

Researchers used EndNote X9.3.3 to remove the duplicate and manage references. Two researchers (P.K. and T.T.) independently screened and identified primary studies based on the inclusion criteria. Researchers used The Johns Hopkins Nursing Evidence‐Based Practice Evidence Rating Scale to appraise the quality of (Dang et al., 2021). Any disagreements between the appraisals were discussed for solutions with the third researcher (P.D.). A collective table was developed with the following fields: author, year, study design, demographic data of study participants, sample size, research instrument, prevalence and factors related. Then, researchers synthesized the narrative synthesis of data in the collective table related to the study aims.

## 3 RESULTS

### 3.1 Study results and identification

**FIGURE 1:**
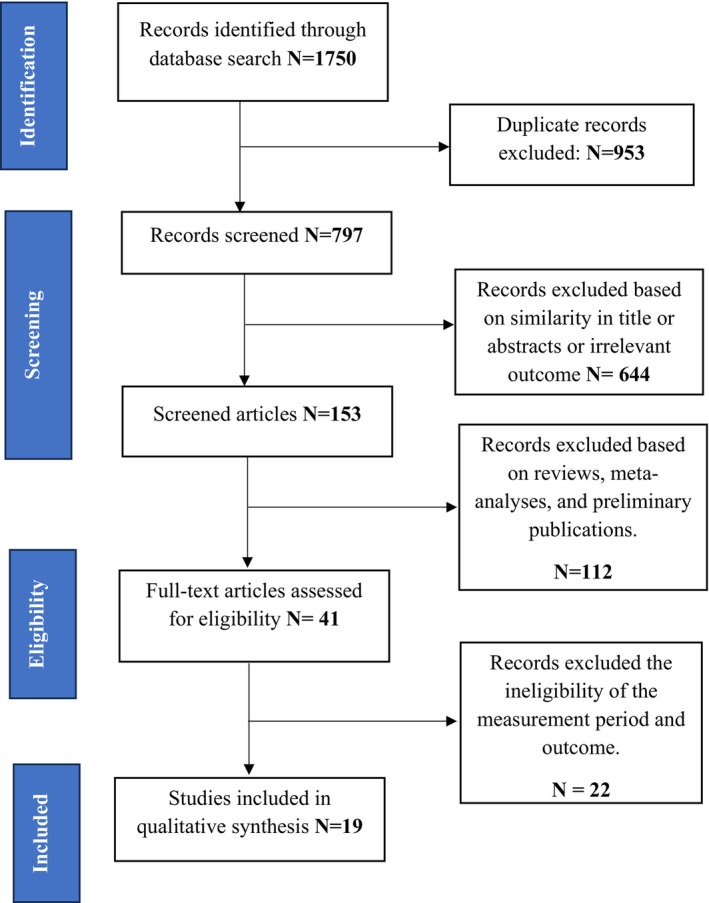
PRISMA (Preferred Reporting Items for Systematic Review and Meta‐Analysis) guideline showing the allocation of evidence for final review.

A total of 1750 articles (164 from PubMed, 279 from EMBASE, 703 were retrieved from Scopus, 69 from Ovid, 205 from MEDLINE and 329 from Science direct). After removing duplicates (*n* = 953), 797 articles remained. Of these, a total of 644 articles entries were removed based on a review of their titles and abstracts. Of the 153 articles screened, a total of 112 articles were excluded due to issues such as reviews, meta‐analyses and preliminary publications. Of the remaining 41 studies, a total of 19 studies that satisfied the inclusion criteria were included in this study. All included evidences were rated level of evidence as level III with quality of evidence as grade A. The procedure of selecting the studies is represented in Figure 1.

### 3.2 Study characteristics

Of the 19 studies, 12 were prospective studies conducted in older adults, aged 60 years and older who underwent surgery with general anaesthesia including hip fracture surgery, non‐cardiac surgery, cardiovascular surgery, radical gastrectomy, colorectal surgery, abdominal surgery, thoracic surgery and other major surgeries. Five studies were retrospective studies of gastrointestinal tumour resection, radical gastric cancer surgery, non‐cardiac surgery and total knee arthroplasty. Two case–control studies were conducted in patients after total hip replacement and non‐cardiac surgery (Table 1).

**TABLE 1:** Predicting factors of POCD in older patients undergoing non‐neurosurgery in immediate postoperative period (*n* = 19 articles).

Author/year	Design	Age (mean)	Sample size	Types of surgery	Instrument	Measurement date (postoperative day)	Prevalence of POCD (%)	Factors associated POCD			Predictors	Estimate effect (OR)	95% CI	*p*‐value
								Preoperative	Intraoperative	Postoperative				
Li et al., 2023	Case–control study	68.5	60	Total hip replacement	Neuro‐psychological battery test	7	35		√	√	‐Surgery time ‐SDNN	2.72 0.91	1.23–7.23 0.83–0.99	0.031 0.034
Zhao et al., 2023	Prospective observational study	70.2	104	Surgery under general anaesthesia.	MMSE	1,3,7	23.1	√		√	‐Smoking ‐NLR ‐PLR	2.01 1.34 1.78	1.11–2.72 1.02–1.79 1.34–2.79	<0.05
Liu et al., 2023	Retrospective study	70.2	196	Non‐cardiac surgery	Neuro‐psychological battery test	7	20.4	√			‐Preoperative LTL	12.33	3.29–46.24	<0.001
Zheng et al., 2023	Prospective observational study	72.2	76	Hip fracture surgery	MoCA MMSE	7	31.6	√		√	‐Education level ‐Preoperative CRP level ‐Postoperative CHI3L1	0.48 1.04 1.20	0.25–0.93 1.00–1.08 1.09–1.33	0.029 0.040 0.001
Huang et al., 2022	Retrospective study	‐	369	Gastrointestinal tumour resection	MMSE	1,7	21.4	√	√		‐Age ‐BMI ‐History of cardiovascular disease ‐Preoperative WBC ‐Blood loss ‐Surgical time	1.72 2.24 3.40 3.75 3.44 6.20	1.00–2.94 1.06–4.74 1.36–8.54 1.12–12.52 1.48–8.00 1.52–25.13	<0.05
Fan et al., 2022	Case–control study	63	60	Non‐cardiac surgery	MMSE	7	34.0			√	‐microRNA‐2213p levels	0.94	0.85–1.00	<0.01
Wang, Wang, et al., 2022	Retrospective study	77	687	Radical gastric cancer surgery	MMSE	7	20.5	√	√		‐Age ‐ASA class ‐Preoperative PG‐SGA score ‐Preoperative haemoglobin ‐Surgery time	3.13 4.67 4.02 4.16 1.33	1.31–8.67 1.61–12.65 1.01–10.88 2.26–8.23 1.03–1.72	0.001 0.020 0.048 0.001 0.027
Wang, Cao, et al., 2022	Prospective cohort study	60	82	Thoraco‐abdominal aortic replacement	Neuro‐psychological battery test	3,7	24.4	√		√	‐Age ‐POD duration ‐sTREM2 level	1.15 2.47 1.06	1.03–1.28 1.15–5.29 1.02–1.11	0.014 0.020 0.009
Lertkovit et al., 2022	Prospective observational study	72.88	250	Elective major surgery	MoCA	5	‐		√		‐Anaesthesia drugs ‐Benzodiazepine ‐Isoflurane	2.24 2.80	1.10–4.68 1.35–5.81	0.006
Ren et al., 2022	Retrospective study	67	103	Total knee arthroplasty (TKA)	MOCA	1,3	‐	√		√	‐Preoperative MOCA score ‐CRP level ‐Pain	2.53 1.03 9.35	1.09–6.06 1.00–1.05 2.54–34.42	0.031 0.038 0.001
Zhao et al., 2021	Prospective observational study	63	75	Cardiovascular Surgery	Neuro‐psychological battery test	7	45.3	√			‐Preoperative LMR level	0.70	0.58–0.82	0.003
Zhang et al., 2021	Retrospective study	66.5	84	Carotid endarterectomy (CEA)	MMSE	2	28.6		√		‐Total intravenous anaesthesia	25.54	2.10–310.6	0.011
Li et al., 2021	Prospective observational study	70.6	222	Abdominal surgery	MMSE	7	41	√	√	√	‐Hypertension ‐Alcohol consumption ‐Preoperative WBC ‐Intraoperative blood loss ‐Pain ‐NLR	3.05 2.40 5.54 3.32 13.82 3.26	1.27–7.32 1.174–4.90 1.13–26.22 1.10–10.06 4.48–39.98 1.02–10.41	0.013 0.016 0.035 0.034 <0.001 0.046
Zhou et al., 2020	Prospective cohort study	72	72	Non‐cardiac surgery	Neuro‐psychological battery test	3	30.56	√	√	√	‐Age ‐Intraoperative blood loss ‐RNA	1.32 1.02 0.94	1.11–1.57 1.00–1.03 0.85–1.00	0.001 0.011 <0.01
Yong & Meng, 2020	Prospective cohort study	71.7	221	Radical gastrectomy	MMSE	7	19	√			‐Preoperative NLR	2.44	1.52–3.68	0.013
Tong et al., 2020	Prospective cohort study	69.8	154	Thoracic Surgery	MoCA	3	49.4	√	√		‐Preoperative MCI ‐Surgery time	0.46 3.79	0.28–0.56 1.37–10.46	<0.001 0.001
Amado et al., 2020	Prospective observational study	65	194	Non‐cardiac surgery	Mini‐Cog test	7	63.9	√			‐Educational level ‐Frailty	3.54 7.54	1.52–8.26 2.02–28.11	0.003
Zhang et al., 2019	Prospective observational study	73.3	80	Colorectal surgery	MMSE	7	24.7	√		√	‐Diabetes ‐Fasting ≥3 days ‐SIRS score >3	2.38 5.24 7.00	1.26–4.51 2.00–13.72 1.95–25.11	<0.05 0.001 0.003
He et al., 2019	Prospective observational study	71.1	124	Gastrointestinal surgery	Neuro‐psychological battery test	7	41.1	√			‐HOMA‐IR value ‐Diabetics	1.88 8.34	1.18–2.99 2.21–31.89	<0.05

Abbreviations: BMI, body mass index; CHI3L1, chitinase‐3‐like‐1 protein; CRP, C‐reactive protein; HOMA‐IR, Homeostatic Model Assessment for Insulin Resistance; LMR, lymphocyte‐to‐monocyte ratio; LTL, leucocyte telomere length; MCI, mild cognitive impairment; MMSE, Mini‐Mental State Examination; MoCA, Montreal Cognitive Assessment NLR, neutrophil–lymphocyte ratio; PG‐SGA, Patient‐Generated Subjective Global Assessment; PLR, platelet/lymphocyte ratio; POD, postoperative delirium; RNA, ribonucleic acid; SDNN, The standard deviation of all R–R intervals.; SIRS, systemic inflammatory response syndrome; sTREM2, soluble triggering receptor expressed on myeloid cells 2; WBC, white blood cell.

### 3.3 Factors predicting for POCD

#### 3.3.1 Preoperative risk factors for POCD in the short‐term postoperative period

Patients' characteristics, such as age, were identified as a risk factor for POCD in four studies in the first week after surgery, with an average age of 70–80 years (Huang et al., 2022; Wang, Cao, et al., 2022; Wang, Wang, et al., 2022; Zhou et al., 2020) and education level was found to be a risk factor for POCD in older adults having only primary school education (Amado et al., 2020; Zheng et al., 2023). Furthermore, five studies reported smoking, alcohol consumption, body mass index (BMI) < 18.5 kg/m^2^, nutritional status (PG‐SGA score ≥4) and frailty (frail score ≥3) were independently associated with POCD (Amado et al., 2020; Huang et al., 2022; Li et al., 2021; Zhao et al., 2023) (Table 1).

Preoperative assessment was reported in 14 studies, preoperative inflammatory biomarkers such as C‐reactive protein (CRP), preoperative white blood cell count (WBC) >10 × 10^9^/L, preoperative neutrophil–lymphocyte ratio (NLR) ≥ 2.50, leucocyte telomere length (LTL) and preoperative lymphocyte‐to‐monocyte ratio (LMR) were found in six studies indicated risk factors for POCD (Huang et al., 2022; Li et al., 2021; Liu et al., 2023; Yong & Meng, 2020; Zhao et al., 2021; Zheng et al., 2023). Two studies reported preoperative assessment including preoperative anaemia and Homeostatic Model Assessment for Insulin Resistance (HOMA‐IR) were associated with cognitive dysfunction (He et al., 2019; Wang, Wang, et al., 2022). In addition, co‐morbidities, such as cardiovascular diseases, diabetes and hypertension, were found in four studies reported those predicted postoperative cognitive decline (He et al., 2019; Huang et al., 2022; Li et al., 2021; Zhang et al., 2019). Moreover, preoperative cognitive score was associated with cognitive dysfunction after surgery (Ren et al., 2022; Tong et al., 2020) (Table 1).

#### 3.3.2 Intraoperative risk factors for POCD in the short‐term postoperative period

Intraoperative risk factors, such as surgery time, was found to be a risk factor of POCD in four studies (Huang et al., 2022; Li et al., 2023; Tong et al., 2020; Wang, Wang, et al., 2022). However, surgical duration depends on type of surgery, in radical gastric cancer surgery found surgery time more than 100 min and thoracic surgery more than 80 min associated cognitive dysfunction (Li et al., 2023; Tong et al., 2020). Older adults undergoing total hip replacement more than 4 h, gastrointestinal tumour resection more than 8 h developed POCD (Huang et al., 2022; Wang, Wang, et al., 2022). Three studies revealed that intraoperative 300 mL in non‐cardiac surgery, more than 400 mL in gastrointestinal tumour resection and intraoperative blood loss more than 500 mL in abdominal surgery independently associated with cognitive dysfunction (Huang et al., 2022; Li et al., 2021; Zhou et al., 2020). Additionally, anaesthesia drug was reported in one study focusing on different types of anaesthesia associated with POCD found that intraoperative benzodiazepine and isoflurane were predictors (Lertkovit et al., 2022). Moreover, total intravenous anaesthesia was an independent risk factor for POCD in carotid endarterectomy patients (Zhang et al., 2021) (Table 1).

#### 3.3.3 Postoperative risk factors for POCD in the short‐term postoperative period

Postoperative inflammatory biomarkers such as soluble triggering receptor expressed on myeloid cells 2 (sTREM2) and CHI3L1 are neuroinflammatory biomarkers; a study found that high levels of sTREM2 and CHI3L1 indicated postoperative cognitive decline (Wang, Cao, et al., 2022; Zheng et al., 2023). Other inflammatory biomarkers including NLR ≥2, platelet/lymphocyte ratio (PLR) higher than preoperative, higher CRP level and plasma microRNA level independently associated POCD (Fan et al., 2022; Li et al., 2021; Ren et al., 2022; Zhao et al., 2023; Zhou et al., 2020). Moreover, finally, SIR score >3 at postoperative day 2 indicated a risk of POCD (Zhang et al., 2019). Two studies reported postoperative pain score (PS ≥4) of resting period postoperative day1 was a statistically significant independent risk factor both in mild and severe cognitive dysfunction (Li et al., 2021; Ren et al., 2022). Moreover, postoperative POD duration ≥2 days was the independent risk factors of POCD (Wang, Cao, et al., 2022). The standard deviation of all R–R intervals <100 ms surgery and fasting period more than 3 day after surgery indicated cognitive dysfunction (Li et al., 2023; Zhang et al., 2019) (Table 1).

## 4 DISCUSSION

This review has identified preoperative, intraoperative and postoperative factors contributing to POCD. The time window for assessment was the first day of the first week after surgery during hospital stay before discharge and the incidence was found to be 22%–41% in this period.

Preoperative risk factors are defined as factors determining in preoperative time; several studies focus on patient‐related factors. Advance age has been previously been shown to be proved to be a crucial risk factor for both short‐term and long‐term cognitive dysfunction in patients with age 70 years and older in a systematic review and meta‐analysis study (Arefayne et al., 2023). The brain of older adults become more vulnerable to anaesthesia and surgery due to age‐related chronic neuroinflammation, abnormal glial cell structure, loss of neurons, impaired synaptic function and dysfunction of the autonomic nervous system (Jiang et al., 2022). Educational level is reported in multiple clinical research have shown lower educational levels as a statistically significant risk factor for POCD (Brown IV & Deiner, 2016). Systematic review and meta‐analysis studies provide evidence that having a lower level of education has been recognized as a risk factor for POCD, whereas having a higher education years serves as a protective factor (Feinkohl et al., 2017).

Inflammatory biomarkers play important roles in pathogenesis of POCD. Several studies explored the biomarkers indicating the incidence and risk factor of POCD; however, it is still unclear. Peripheral inflammation can change the structure and function of the blood–brain barrier, leading to a decrease in synaptic plasticity and neuronal cell death, which impairs the restoration of cognitive function after surgery (Jin et al., 2020). In this review we found that preoperative inflammatory biomarkers, including the LTL, NLR, LMR, CRP and WBC, associated with POCD. Research has shown that even bare elevation in WBC count within the typical range are strongly linked to cognitive decline and NLR was statistically significant in MCI (An et al., 2019; Kao et al., 2011). Moreover, the potential new biomarker for measuring inflammatory response has been identified as LMR a composite of two independent indicators of inflammation which may progress to neuroinflammation (Umehara et al., 2020). CRP is an indicator of a general acute reaction in inflammation, infection and tissue injury that is associated with cognitive dysfunction (Pepys & Hirschfield, 2003). This review found that longer LTL is a strong predictor of POCD. Multiple studies have consistently found that individuals with longer LTL values tend to have lower cognitive abilities on tests measuring functional language fluency and case memory (Harris et al., 2006).

Comorbidities such as hypertension, diabetes and cerebrovascular disease were predictors of POCD that were found in abdominal surgery. The study provides evidence that hypertension is additionally linked to the accumulation of the beta‐amyloid peptide (Perrotta et al., 2016). There is a recent connection between low levels of beta amyloid in cerebrospinal fluid, which indicates the initial stages of Alzheimer's disease, and the occurrence of POCD (Evered et al., 2016). People diagnosed with diabetes and elevated blood glucose levels are more prone to have POCD. Diabetes contributes to vascular abnormalities leading to a continuous decrease in blood supply to the brain. As a result, there is a continuous reduction in the supply of blood to the brain, leading to anomalies in the brain's white matter. The brain's capacity to withstand alterations is diminished as a result (Nakao et al., 2019).

Additionally, frailty alters cognitive function by increasing neurodegeneration resulting in chronic inflammation and oxidative stress. The body system promotes the increasing of proinflammatory cytokines such as IL‐6, TNF‐α which relate with physical frailty condition. Oxidative stress causes the mitochondrial dysfunction resulting in the increasing production of reactive oxygen species which associates with physical frailty (de Morais Fabrício et al., 2020). Malnutrition was found to be related with cognitive impairment because of the limited neuroprotective food intake, particularly Mediterranean diet. Neuroprotective food could prevent the brain damage from brain inflammation and oxidative stress (Feng et al., 2022). Smoking and alcohol drinking were also independent risk factors in this review. Nicotine stimulates endothelial cells via binding to nicotinic acetylcholine receptors located on the endothelium. This activation leads to an increase in pathological angiogenesis (Wang et al., 2021).

Intraoperative risk factors are factors that occur during operative time including anaesthetic and surgical factors associated with declining cognitive function after surgery. An extended duration of the surgical procedure would exacerbate the adverse effects produced by an inadequate flow of blood to the brain, thereby impacting memory and cognitive function after the operation (Taylor et al., 2022). Excessive bleeding during surgery and dropping of haemoglobin concentration, caused by an inflammatory response and damage to the blood–brain barrier, can contribute to POCD (Taylor et al., 2022). Isoflurane is an inhalation anaesthetic that is a necessary component for sustaining general anaesthesia for extended durations, although it can have negative effects on the cognitive abilities of older individuals. However, there have been few studies undertaken in humans that have examined the connection between specific inhalation agents and the incidence of POCD (Belrose & Noppens, 2019).

Postoperative factors are factors that occur after surgery associated with POCD. Inflammatory biomarkers after surgery such as NLR, PLR and CRP level are independent risk factors of POCD in immediate phase. Biomarkers are novel inflammatory indicators that have been used more frequently in recent years for the quick identification, early diagnosis and prognosis evaluation of various diseases (Cupp et al., 2020). Postoperative pain might worsen POCD via increasing level of dopamine in the cortex and decreasing level acetylcholine in the hippocampus, and altering the levels of inflammatory substances (Ding et al., 2021). Delirium after operation leads to alterations in the structural integrity of white matter and damage to the subcortical region of the brain. These changes subsequently have a detrimental impact on the cognitive processing speed of older individuals who have undergone surgery, resulting in POCD (Brown IV et al., 2018). A systematic review found that postoperative patients who experience POD have been found to be at higher risk of POCD (Arefayne et al., 2023).

## 5 STRENGTHS AND LIMITATIONS

The strength of this systematic review includes the emphasis on the importance of POCD in older populations undergoing non‐neurosurgery because the cognitive function was rarely examined and managed in this group of populations. Then, researchers identified the factors related to POCD in each phase of the operation. However, there were some limitations of this study. Researchers could not develop the meta‐analysis to identify the effect of each factor with outcomes due to the heterogeneity of literature. In addition, we included literature published only in English so various studies in other languages were not included in this study.

## 6 CONCLUSION

We identified several independent risk factors and incidences of POCD in older patients undergoing non‐neurosurgery in the immediate phase after surgery in recent years classified by the phase of operation. The factors related with POCD in each phase were as follows: (1) preoperative risk factors found age, educational attainment, malnutrition, preoperative biomarkers and co‐morbidities; (2) intraoperative risk factors were the duration of operation, blood loss during operation and anaesthesia used; and (3) postoperative risk factors consisted of postoperative biomarkers and postoperative pain. Consequently, the review addresses the importance of POCD management researchers and healthcare providers should monitor the underlying causes and screen the risk factors of POCD, and in formulating suitable preventative and therapeutic strategies.

## AUTHOR CONTRIBUTIONS

Pinyapat Kitthanyateerakul developed the study concept, conducted literature searching, screening and appraisal, and wrote the manuscript. Thitipong Tankumpuan conducted literature searching, screening and appraisal, and wrote the manuscript. Patricia Davidson wrote the manuscript and reviewed the formatting and completion of tables and figures.

## FUNDING INFORMATION

This research received no specific grant from any funding agency in the public, commercial or not‐for‐profit sectors.

## CONFLICT OF INTEREST STATEMENT

The authors declare no conflicts of interest.

## ETHICS STATEMENT

No Research Ethics Committee approval was needed.

## Supporting information


Data S1.

## Data Availability

The data that support the findings of this study are available from the corresponding author upon reasonable request.
